# Proof of anthocyanins in the carnivorous plant genus *Nepenthes*

**DOI:** 10.1002/2211-5463.13255

**Published:** 2021-08-04

**Authors:** Alberto Dávila‐Lara, Michael Reichelt, Ding Wang, Heiko Vogel, Axel Mithöfer

**Affiliations:** ^1^ Research Group Plant Defense Physiology Max Planck Institute for Chemical Ecology Jena Germany; ^2^ Department of Biochemistry Max Planck Institute for Chemical Ecology Jena Germany; ^3^ Department of Insect Symbiosis Max Planck Institute for Chemical Ecology Jena Germany

**Keywords:** anthocyanins, betalain, Caryophyllales, *Nepenthes*, plant carnivory

## Abstract

Yellow to red colored betalains are a chemotaxonomic feature of Caryophyllales, while in most other plant taxa, anthocyanins are responsible for these colors. The carnivorous plant family Nepenthaceae belongs to Caryophyllales; here, red‐pigmented tissues seem to attract insect prey. Strikingly, the chemical nature of red color in *Nepenthes* has never been elucidated. Although belonging to Caryophyllales, in *Nepenthes*, some molecular evidence supports the presence of anthocyanins rather than betalains. However, there was previously no direct chemical proof of this. Using ultra‐high‐performance liquid chromatography‐electrospray ionization‐high‐resolution mass spectrometry, we identified cyanidin glycosides in *Nepenthes* species and tissues. Further, we reveal the existence of a complete set of constitutively expressed anthocyanin biosynthetic genes in *Nepenthes*. Thus, here we finally conclude the long‐term open question regarding red pigmentation in Nepenthaceae.

## Graphical abstract

Red colors are widely distributed in tissues of carnivorous *Nepenthes* plants. However, whether these colors are due to the presence of anthocyanins or betalains remained unclear. Here, we employed ultra‐high‐performance liquid chromatography‐electrospray ionization‐high‐resolution mass spectrometry and identified different cyanidin glycosides in *Nepenthes* species and tissues. In addition, the existence of a complete set of constitutively expressed anthocyanin biosynthetic genes in *Nepenthes* is shown.

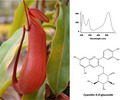

**Abbreviations**
ADH: arogenate dehydrogenase
ANS: anthocyanidin synthase
C3′H: 5-*O*-(4-coumaroyl)-D-quinate 3′-monooxygenase
CHI: chalcone isomerase
CHS: chalcone synthase
DFR: dihydroflavonol 4-reductase/flavonone 4-reductase
DODA: DOPA 4,5-dioxygenase
DOPA: 3,4-dihydroxyphenylalanine
ESI-MS/MS: electrospray ionization with tandem mass spectrometry
F3′5′H: flavonoid 3′,5′-hydroxylase
F3′H: flavonoid 3′-monooxygenase
F3H: flavanone 3-dioxygenase
HCT: shikimate *O*-hydroxycinnamoyl transferase
UFGT: anthocyanidin 3-*O*-glucosyltransferase
UHPLC–ESI–HRMS: ultra-high-performance liquid chromatography-electrospray ionization-high-resolution mass spectrometry

The presence of betalains is a typical phytochemical feature of the plant order Caryophyllales [1]. Betalains are violet to red (betacyanins) and orange to yellow (betaxanthins) pigments that are derived from the amino acid tyrosine. According to the Angiosperm Phylogeny Group classification [2], the occurrence of betalains holds true for the so‐called core Caryophyllales, a defined clade of eudicots comprising *c*. 29 families. Only two Caryophyllales families, Caryophyllaceae and Molluginaceae, do not contain betalains but anthocyanins, also red and yellow pigments much more widely distributed in the plant kingdom [3, 4]. Within the noncore Caryophyllales, betalains have never been documented. Strikingly, the presence of betalains and anthocyanins exclude each other [1, 3, 5, 6]. Very likely, this is due to two events in the core Caryophyllales, the de‐regulation of the tyrosine biosynthesis and gene duplication events [3, 6, 7]. While betalains are made from tyrosine, anthocyanins are made from phenylalanine. In both cases, the shikimate pathway provides the biosynthetic precursors; that is, the syntheses of tyrosine and phenylalanine compete for prephenate or arogenate as substrate. Whereas during tyrosine synthesis in bacteria and fungi prephenate is converted to 4‐hydroxyphenyl pyruvate, in plants prephenate is mainly converted to arogenate. Arogenate is further converted by arogenate dehydrogenases (ADH) into tyrosine. Typically, the ADH is negatively feedback‐regulated by tyrosine; however, in betalain accumulating species this regulation is partly lost. Here, a tyrosine‐insensitive ADH arose during evolution of the betalain synthetic pathway in the core Caryophyllales that accumulate high amounts of tyrosine and, as a consequence thereof, the substrate for betalain synthesis [7]. In addition, two enzymes downstream of tyrosine synthesis, CYP76AD1 and a 3,4‐dihydroxyphenylalanine (DOPA) 4,5‐dioxygenase (DODA), underwent gene duplication and concomitant neo‐functionalization. These duplications gave rise to DODA‐α and CYP76AD1‐α isoforms, which seem necessary for betalain synthesis; both new genes arose shortly before the origin of betalain pigmentation [3].

Nepenthales were classified as a noncore group of Caryophyllales [8], which are characterized among other features by lacking betalains [9]. Nepenthales cover noncarnivorous as well as carnivorous lineages. The latter lineage consists of five plant families, including Droseraceae with the genera *Drosera* (sundew) and *Dionaea*, represented by the only species *Dionaea muscipula* (Venus flytrap), and Nepenthaceae with the genus *Nepenthes* (pitcher plants). Almost all of these carnivorous plants have parts with intensive red colors, which are potentially involved in prey attraction. Maybe due to the classification into core and noncore Caryophyllales, there is still confusion about the nature of the red color in carnivorous plants belonging to Caryophyllales. While in *Drosera* spp. anthocyanins, cyanidin and pelargonidin glycosides, have been strongly suggested [5, 10], for *D*. *muscipula* the presence of cyanidin‐3‐glucoside was demonstrated already in 1966 by chromatographic and spectroscopic (UV, IR) methods in comparison with an authentic standard [11]. Recently, a combination of spectrophotometry, HPLC co‐elution and electrospray ionization with tandem mass spectrometry (ESI‐MS/MS) proved the presence of delphinidin‐3‐*O*‐glucoside (myrtillin), cyanidin‐3‐*O*‐glucoside (kuromanin), and the cyanidin aglycone in *D*. *muscipula* [12].

For the genus *Nepenthes*, the situation is not as clear as for *Drosera* and *Dionaea*. There is still no evidence for anthocyanin presence in *Nepenthes*. Nevertheless, there are speculations about the nature of the red coloration in this genus, but no robust data yet. The optical properties of anthocyanins and betalains are very similar, and simple UV/Vis absorption measurements at, for example, 532 nm [13] cannot discriminate both compounds and does not really justify a statement on the pigment's nature. Moreover, some citations are inaccurate and, thus, mere suggestions seem to become true the more often a reference is cited. For example, Moran and Moran [14] are repeatedly cited for the presence of anthocyanins [15, 16]. However, with the foliar reflectance analysis used in that study it was not possible to really prove the presence of anthocyanins as the spectral data of anthocyanin and betacyanin are very similar [17]. Also, the study of [18] was listed as a reference for anthocyanins [19] although they only analyzed phenolic compounds and flavonols.

Nevertheless, it has been common sense for many years that also *Nepenthes* species contain anthocyanins, not at least due to molecular studies [1, 3, 6, 7, 20]. Unfortunately, the final proof based on reliable chemical analytics as in the case of *Dionaea* [12] is still missing for *Nepenthes*. Here, we aim to evaluate the presence of anthocyanins in *Nepenthes* tissues by employing sensitive analytical techniques, that is, ultra‐high‐performance liquid chromatography–ESI–high‐resolution mass spectrometry (UHPLC–ESI–HRMS). We detected three different cyanidin derivatives and, moreover, found all genes that are necessary for the anthocyanin biosynthetic pathway constitutively expressed.

## Materials and methods

### Plant material

*Nepenthes x ventrata* (the natural hybrid of *Nepenthes* *ventricosa* × *Nepenthes* *alata*), *Nepenthes* *thorelii, N. ventricosa* plants were grown in the MPI greenhouse at 21–23 °C, 50–60% relative humidity and a 16 h light/8 h dark photoperiod. To keep the plants moistened, they were sprayed with distilled water for 25 s four times per day. Pitchers from *Nepenthes* *robcantleyi, Nepenthes* *maxima, Nepenthes* *fusca*, and *Nepenthes* *mirabilis* were provided from the Botanical Garden, Jena, Germany.

Matured and well‐developed pitchers were sampled from different plants representing independent biological replicates. Digestive fluid from pitcher was discarded, and pitcher was rinsed three times with sterile ddH_2_O. Afterward, tissues of interest (peristome, digestive zone, leaf, branches) were sampled and directly frozen in liquid N_2_.

### Extraction and quantification of anthocyanins by HPLC‐UV

Frozen tissue samples were ground and 100 mg fresh weight powder extracted with 1.0 mL ddH_2_O:MeOH (50 : 50 v/v). After mixing, samples were sonicated for 15 min on ice‐cold water bath. Therefore, shook for 30 min at 4 °C using Rotator Mixer RM‐Multi‐1 (STARLAB GmbH, Hamburg, Germany) with the following settings: orbital at 100 r.p.m. for 15 s, reciprocal at 75° for 16 s, and vibro at 3° for 5 s. Samples were centrifuged afterward at 16 000 ***g*** at 4 °C for 30 min, and clear supernatants were collected and used for further analysis.

Anthocyanins were analyzed by reversed‐phase HPLC with UV detection using an Agilent 1100 system (Agilent Technologies, Waldbronn, Germany): column used: Nucleodur Sphinx RP (250 × 4.6 mm, 5 µm; Macherey‐Nagel, Düren, Germany); injection volume was 50 μL; flow rate, 1.0 mL·min^−1^; solvent A, 0.5% (v/v) trifluoroacetic acid; solvent B, acetonitrile. The photodiode array detector was used in the range of 250–650 nm. Samples were analyzed with the following chromatographic gradient: start 5% B, linear gradient from 5% B to 25% B in 20 min followed by a washing cycle. Peaks at 18.1 min and at 18.5 min in the HPLC‐UV/Vis chromatograms were identified by match of retention time with commercial standards as cyanidin‐3‐*O*‐galactoside (Extrasynthese, Genay, France) and as cyanidin‐3‐*O*‐glucoside (TransMIT GmbH, Gießen, Germany), respectively. Further identification is based on LC‐ESI‐HRMS (see below). Quantification was achieved by detection at 520 nm using a calibration curve generated from authentic cyanidin‐3‐*O*‐glucoside.

### Identification of anthocyanins by LC‐ESI‐HRMS

Chemical structures of anthocyanins were determined by UHPLC–ESI–HRMS performed with a Dionex Ultimate 3000 series UHPLC (Thermo Scientific, Schwerte, Germany) and a Bruker timsToF mass spectrometer (Bruker Daltonics, Bremen, Germany). UHPLC was used applying a Zorbax Eclipse XDB‐C18 column (100 mm × 2.1 mm, 1.8 µm; Agilent Technologies) with a solvent system of 0.1% (v/v) formic acid (A) and acetonitrile (B) at a flow rate of 0.3 mL·min^−1^. The elution profile was the following: 0–0.5 min, 5% B; 0.5–11.0 min, 5–60% B; 11.0–11.1 min, 60–100% B, 11.1–12.0 min, 100% B and 12.1–15.0 min 5% B. ESI in positive ionization mode was used for the coupling of LC to MS. The mass spectrometer parameters were set as follows: capillary voltage 4.5 KV, end plate offset of 500 V, nebulizer pressure 2.8 bar, nitrogen at 280 °C at a flow rate of 8 L·min^−1^ as drying gas. Acquisition was achieved at 12 Hz with a mass range from *m*/*z* 50 to 1500 with data‐dependent MS^2^. Fragmentation was triggered at the two most intense peaks applying a target intensity of 20 000 counts, with MS^2^ spectra acquisition at 2 Hz, and a limited total cycle time of 2 s. Collision energy was alternated between 20 and 50 to achieve mixed MS^2^ spectra.

### Search for betalains by HPLC‐UV and LC‐ESI‐HRMS

The HPLC‐UV chromatograms at 520 nm from quantification of anthocyanins (see above) where searched for additional peaks that might correspond to betalains. However, in the HPLC‐UV chromatograms at 520 nm no other peaks apart from the three described anthocyanins were found. Additionally, betalains were searched for in the raw data from the LC‐ESI‐HRMS runs in positive ionization mode described above for structure elucidation of anthocyanins. Extracted ion chromatograms for the molecular ion peak [M + H]^+^ of known betalains [21, 22] with an isolation width of *m*/*z* 0.002 (Table 1) were inspected for possible peaks. For none of the tested known betalains an [M + H]^+^ peak could be detected, this means the compound(s) are not there or below the detection limit of the LC‐ESI‐HRMS system.

**Table 1:** Betalains searched in *Nepenthes* spp. peristomes by LC‐ESI‐HRMS.

Compound	Molecular sum formula	Theoretical *m*/*z* for [M + H]^+^ molecular ion	Detected (D)/not detected (ND)
Amaranthine	C_30_H_34_N_2_O_19_	727.182853	ND
Isoamaranthine	C_30_H_34_N_2_O_19_	727.182853	ND
Iresinin I	C_36_H_42_N_2_O_23_	871.225112	ND
Isoorientin I	C_36_H_42_N_2_O_23_	871.225112	ND
Celosianin I	C_39_H_40_N_2_O_21_	873.219633	ND
Isocelosianin I	C_39_H_40_N_2_O_21_	873.219633	ND
Celosianin II	C_40_H_42_N_2_O_22_	903.230197	ND
Isocelosianin II	C_40_H_42_N_2_O_22_	903.230197	ND
Gomphrenin I	C_24_H_26_N_2_O_13_	551.150765	ND
Isogomphrenin I	C_24_H_26_N_2_O_13_	551.150765	ND
Gomphrenin II	C_33_H_32_N_2_O_15_	697.187545	ND
Isogomphrenin II	C_33_H_32_N_2_O_15_	697.187545	ND
Gomphrenin III	C_34_H_34_N_2_O_16_	727.198110	ND
Isogomphrenin III	C_34_H_34_N_2_O_16_	727.198110	ND
Betanin	C_24_H_26_N_2_O_13_	551.150765	ND
Isobetanin	C_24_H_26_N_2_O_13_	551.150765	ND
Betanidin	C_18_H_16_N_2_O_8_	389.097942	ND
2‐Descarboxy‐betanidin	C_17_H_16_N_2_O_6_	345.108113	ND
Lampranthin II	C_34_H_34_N_2_O_16_	727.198110	ND
3‐Methoxytyramine‐betaxanthin	C_18_H_20_N_2_O_6_	361.139413	ND
(*S*)‐Tryptophan‐betaxanthin	C_20_H_19_N_3_O_6_	398.134662	ND
Indicaxanthin	C_14_H_16_N_2_O_6_	309.108113	ND
Miraxanthin‐V	C_17_H_18_N_2_O_6_	347.123763	ND
Betalamic Acid	C_9_H_9_NO_5_	212.055349	ND

### Transcriptome analysis: sampling, total RNA extraction, cDNA library preparation, and sequencing

For transcriptome analysis, *N. x* *ventrata* pitchers were collected first at the opening day of the lid as a reference time point and at the next two consecutive days. To avoid contamination, still‐closed pitchers were covered with a mesh as described in [23]. Pitchers were rinsed three times with sterile ddH_2_O, digestive zone tissue was dissected sampled in 50 mL polypropylene tubes and directly frozen in liquid N_2_. Individual pitchers represent independent biological replicates from different plants. A total of 12 biological replicates were used for RNAseq analysis, with four replicates for each time point.

Dissected digestive zones material was finely ground in liquid N_2_ using mortar and pestle. Samples were stored at −80 °C until RNA extraction was performed. A 50 mg weighed powdery tissue material was used for total RNA isolation. The extraction was done at room temperature using RP InviTrap® Spin Plant RNA Mini kit (STRATEC Molecular, Berlin, Germany) according to the manufacturer's protocol with some modifications. Total RNA was dissolved in ddH_2_O. Each biological sample (digestive zone) was extracted from seven technical replicates and pooled in the final step of RNA elution.

For assessing a rough indicator of quality and yield, A260/A280 and A260/A230 ratios for RNA preparation samples were determined with NanoDrop UV/Vis Spectrophotometer (Thermo Fisher, Schwerte, Germany). To remove any DNA contamination, the isolated RNA was treated with Turbo DNA‐free Kit™ (Invitrogen™, Darmstadt, Germany).

Finally, sample quality control was performed using the yield and the assessment of RNA integrity number. This was done based on comparative evaluation of 28S/18S rRNA on an Agilent 2100 Bioanalyzer system following manufacturer's protocol and performed on an Agilent RNA 6000 Nano LabChip® Kit (Agilent Technologies). Transcriptome sequencing was carried out by the Max Planck Genome Center (Cologne, Germany) (https://mpgc.mpipz.de/home/) using poly(A)+ enriched RNA fragmented to an average of 180 nucleotides. Sequencing was done on an Illumina HiSeq3000 Genome Analyzer platform, using standard TruSeq procedures and paired‐end (2 × 150 bp) read technology, yielding approximately 15 million reads for each of the 28 *N. x* *ventrata* samples.

### Transcriptome assembly, mapping, and annotation

Quality control measures and *de novo* transcriptome assembly, using the combined RNAseq sequence data was carried out using CLC Genomics Workbench v11.1 (http://www.clcbio.com) To assess transcriptome completeness, we performed a BUSCO (Benchmarking Universal Single‐Copy Orthologs; http://busco.ezlab.org) analysis by comparing our assembled transcript sets against a set of highly conserved single‐copy orthologs. This was accomplished using the BUSCO v3 pipeline [24], comparing the predicted proteins of the *N. x* *ventrata* transcriptome to the predefined set of 1614 Embryophyta single‐copy orthologs from the OrthoDB v9.1 database. This resulted in 78.7% complete/partial and 21.3% missing BUSCO genes for the pitcher transcriptome assembly. Digital gene expression analysis was carried out using CLC Genomics Workbench v9.1 to generate BAM (mapping) files, and qseq Software (DNAStar Inc., Madison, WI, USA) was then used to estimate gene expression levels. Sequence similarity searches of the transcriptome were performed using the NCBI BLAST suite on a Galaxy server against the NCBI nr database. Further sequence annotations were done using Gene Ontology (GO) and InterPro terms (InterProScan, EBI), enzyme classification (EC) codes, and metabolic pathways (Kyoto Encyclopedia of Genes and Genomes, KEGG) implemented in BLAST2GO v5.2 (https://www.biobam.com).

### KEGG pathway enrichment analysis

Based on the KEGG orthology (KO), 10 enzymes involved in the anthocyanin biosynthetic pathway were identified: chalcone synthase (K00660); chalcone isomerase (K01859); flavanone 3‐dioxygenase (K00475); flavonoid 3′‐monooxygenase (K05280); flavonoid 3′,5′‐hydroxylase (K13083); bifunctional dihydroflavonol 4‐reductase/flavonone 4‐reductase (K13082); anthocyanidin synthase, (K05277); anthocyanidin 3‐*O*‐glucosyltransferase (K12930); shikimate *O*‐hydroxycinnamoyl transferase (K13065); 5‐*O*‐(4‐coumaroyl)‐d‐quinate 3′‐monooxygenase (K09754). To verify the putative proteins, all sequences were searched via blastx (*e*‐value 1e‐3) against NCBI nonredundant database (available from: https://www.ncbi.nlm.nih.gov/; April 23, 2021). Focusing on the essential enzymes, dominantly expressed transcripts with log_2_RPKM > 2 (RPKM: reads per kilo base per million mapped reads) at least in one out of three independently on three consecutive days harvested *N. x* *ventrata* pitcher samples were selected in this study (Table S1).

## Results and Discussion

Anthocyanins are a water‐soluble group of plant pigments derived from flavonoids. These pigments are produced in the central vacuole of plant cells and occur in reproductive (flowers and fruits) or vegetative (stems, roots, or leaves) plant organs [25]. Still, in vegetative tissues the functional role of anthocyanins remains a controversial topic, ranging from stress response to drought and nutrient deficiency, photoprotection, free radical scavenging, and herbivory defense [26]. In the taxon of core Caryophyllales, anthocyanins are replaced by betalains and the presence of one of these pigments excludes that of the other [1, 3, 5, 6]. Betalains, therefore, have been seen and used as chemotaxonomic markers [27]. Due to their similar locations in plant tissues and cells and their comparable optical features, both pigments are considered to be functional homologues in plant environment interactions [28].

**Fig. 1:**
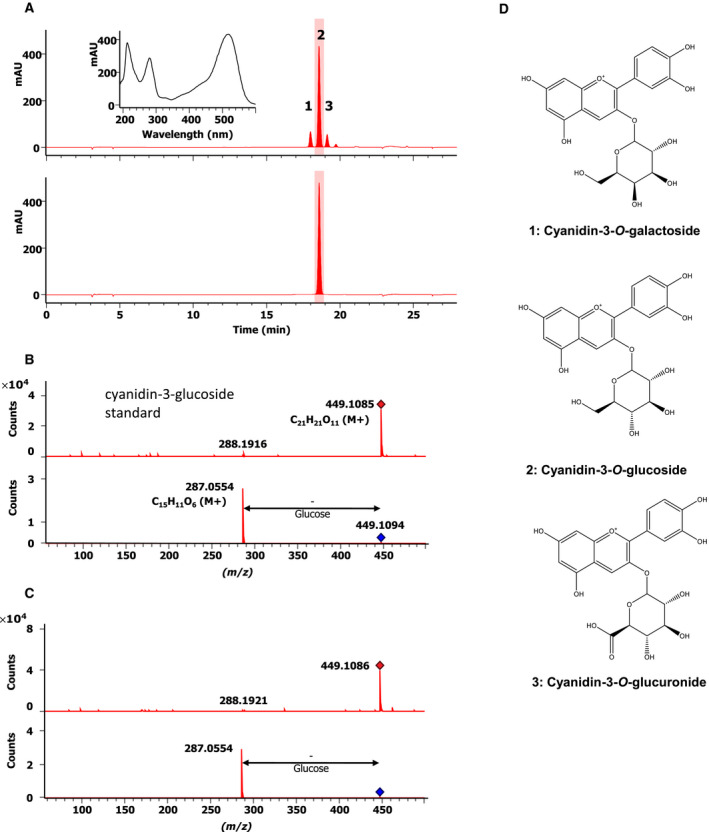
Identification of cyanidin‐3*‐*O‐glycosides as the major anthocyanidin compounds in *Nepenthes x ventrata* peristome tissue. (A) HPLC‐UV/Vis chromatograms at 520 nm for a *N. x* *ventrata* peristome extract (upper chromatogram) and a cyanidin‐3‐*O*‐glucoside standard (lower chromatogram). Insert: UV spectrum of (peak #2). Peak #1 was identified as cyanidin‐3‐*O*‐galactoside by comparison to a commercial standard. Peak #3 was tentatively identified by HRMS as cyanidin‐3‐*O*‐glucuronide. (B) Full scan HR‐MS spectrum (positive mode) and MS^2^ fragmentation spectrum for cyanidin‐3‐*O*‐glucoside standard and (C) peak #2 of *N. x* *ventrata* peristome extract. (D) Structures of anthocyanins identified from *N. x* *ventrata* peristome extract. 1. Cyanidin‐3‐*O*‐galactoside corresponding to peak #1 in (A); Chemical formula: C_21_H_21_O_11_^+^; 2. Cyanidin‐3‐*O*‐glucoside corresponding to peak #2 in (A); Chemical formula: C_21_H_21_O_11_^+^; 3. Cyanidin‐3‐*O*‐glucuronide corresponding to peak #3 in (A); Chemical formula: C_21_H_19_O_12_^+^.

Carnivorous plants of the Droseraceae and Nepenthaceae have been usually assigned to the Caryophyllales *sensu stricto*, but not to the core group of this order. Thus, for a long time it was not clear which pigments occur in these plants. While for *D*. *muscipula* (Droseraceae) a recent investigation by Henarejos‐Escudero *et al*. [12] eventually demonstrated the presence of the anthocyanins delphinidin‐3‐*O*‐glucoside and cyanidin‐3‐*O*‐glucoside, as well as the aglycone cyanidin, thereby confirming earlier but from a chemical point of view less conclusive results; for Nepenthaceae such study was still missing. As shown in Fig. 1, for *N. x* *ventrata* peristomes there are only three peaks detected in an HPLC‐UV analysis at 520 nm, all three showing the typical UV spectrum of anthocyanins (see inset Fig. 1A). By match of retention time and UV spectra with commercial standards, we proved the presence of cyanidin‐3‐*O*‐glucoside (Fig. 1A, peak #2, major peak; Fig. 1D) and cyanidin‐3‐*O*‐galactoside (Fig. 1A, peak #1; Fig. 1D). The identity was further supported by analysis by LC‐ESI‐HRMS (Fig. 1C), the molecular ion peak ([M + H]^+^) of peak #2 at *m*/*z* 449.1085 fits the molecular formula of C_21_H_21_O_11_(Δ −1.47 p.p.m.), the fragment by collision‐induced dissociation at *m*/*z* 287.0554 suggests a cyanidin aglycone structure (C_15_H_11_O_6_, Δ −1.34 p.p.m.). The analysis of the commercial cyanidin‐3‐*O*‐glucoside standard resulted in almost identical values (Fig. 1B). The full scan MS data and MS^2^ fragmentation pattern data strongly suggest the presence of cyanidin‐3‐*O*‐glucuronide (*m*/*z* 463.0874, C_21_H_19_O_12_, Δ −0.64 p.p.m.; Fig. 1D), fragment after CID: *m*/*z* 287.0553, C_15_H_11_O_6_, Δ −0.99 p.p.m.), represented as peak #3 in the HPLC‐UV/Vis chromatogram at 19.2 min (Fig. 1A). These results represent the missing proof for the existence of anthocyanins in Nepenthaceae.

**Fig. 2:**
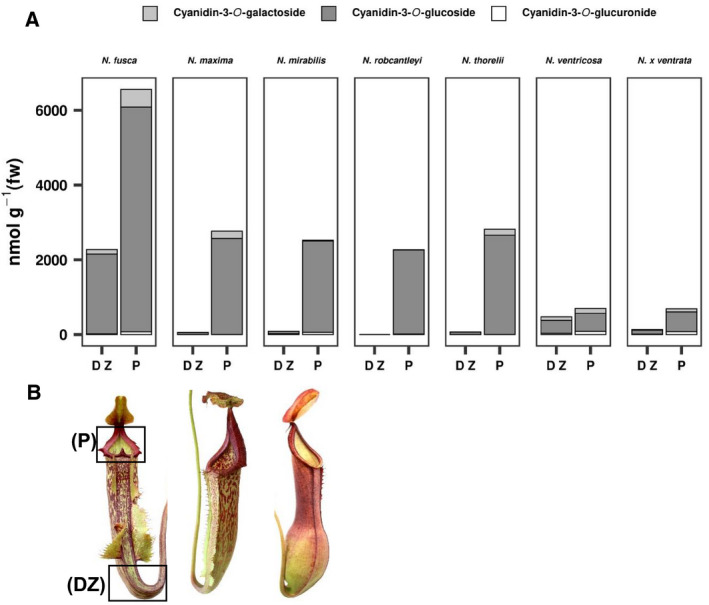
Proof of principle for the presence of anthocyanins in various species of the genus *Nepenthes*. (A) Distribution and relative amounts of different cyanidin anthocyanins in peristomes and digestive zones of seven *Nepenthes* species. (B) Pitcher tissue pigmentation in three *Nepenthes* species (*Nepenthes* *fusca, Nepenthes* *maxima*, and *Nepenthes* *mirabilis*). Black boxes show dissected pitcher tissues harvested for anthocyanin quantification (DZ, digestive zone; P, peristome).

We next analyzed the red colored peristomes and digestive zone of the pitcher of six additional *Nepenthes* species growing from Philippines (*N. x* *ventrata*; *N*. *ventricosa*; *N*. *robcantleyi*, endemic on the island Mindanao), Borneo (*N*. *fusca*), Sulawesi (*N. maxima*), Vietnam (*N*. *thorelii*), all together representing the huge area of Southeast Asia. In all species, the dominating anthocyanin was cyanidin‐3‐*O*‐glucoside, followed by cyanidin‐3‐*O*‐galactoside and cyanidin‐3‐*O*‐glucoronide. In all cases, the peristome contained more anthocyanins than the digestive zone, which matches well with the red color of the tissues (Fig. 2). Within the different species, the highest anthocyanin concentration was found in *N*. *fusca* (*c*. 6.56 µmol·g^−1^ fresh weight in peristome and 2.27 µmol·g^−1^ fresh weight in digestive zone, respectively). The lowest peristome concentrations were determined in *N*. *ventricosa* and *N. x* *ventrata* both with 0.68 µmol·g^−1^ fresh weight, the lowest digestive zone concentration in *N. maxima, N. mirabilis*, and *N*. *thorelii* with less than 0.1 µmol·g^−1^ fresh weight. In *N*. *robcantleyi*, no anthocyanins at all could be detected in the digestive zone (Fig. 2). These results indicate that the presence of anthocyanins is widely distributed within Nepenthaceae but also that the different species contain different levels of these pigments.

**Fig. 3:**
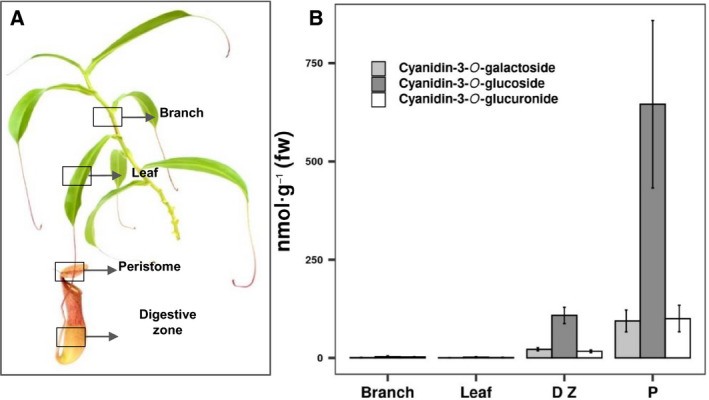
Distribution and concentrations of anthocyanins in *Nepenthes x ventrata* tissues. (A) Indication of four tissues analyzed for anthocyanin presence: branch, leaf blade, digestive zone, peristome. (B) Determined concentrations of different anthocyanins in *N. x* *ventrata* tissues (*n* = 3; mean ± SEM).

A more detailed, tissue‐specific anthocyanin analysis was performed with branches, leaf blades, pitcher digestive zone, and peristomes of *N. x* *ventrata* (Fig. 3A). While in the nonred branch and leaf tissues almost no anthocyanins could be found, both the digestive zone and the peristome tissues contained mainly cyanidin‐3‐*O*‐glucoside; the level of cyanidin‐3‐*O*‐galactoside and cyanidin‐3‐*O*‐glucoronide were similar (Fig. 3B). The ratio between the three anthocyanins were constant in the different tissues.

**Fig. 4:**
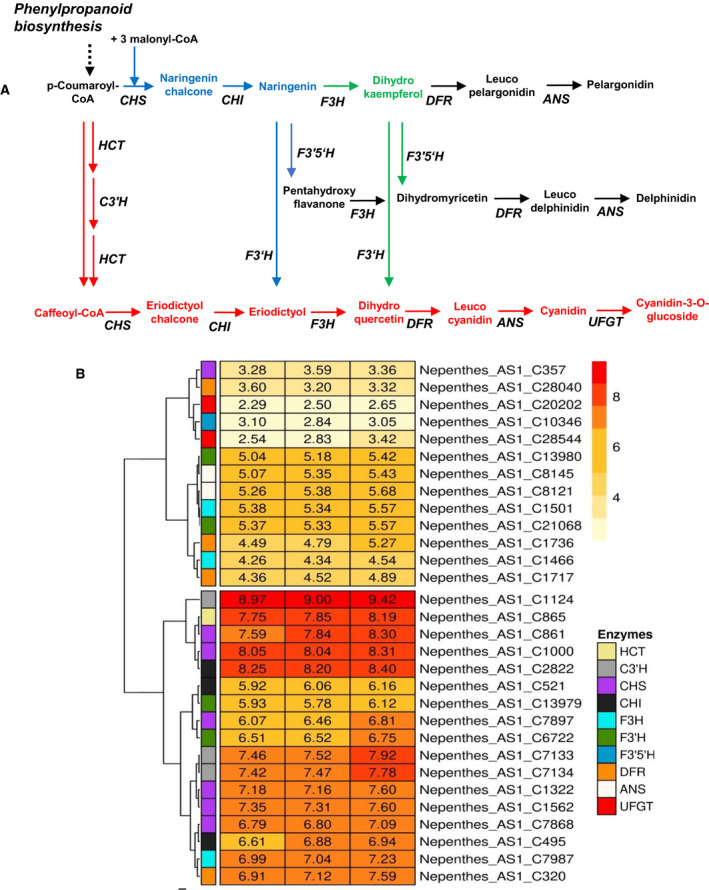
Molecular evidence for anthocyanin biosynthetic pathways in *Nepenthes x ventrata* pitchers. (A) Anthocyanin biosynthesis pathways. Red, blue, and green colors represent putative pathways for cyanidin 3‐*O*‐glucoside synthesis in *N. x* *ventrata*. In black, the metabolic pathway for pelargonidin and delphinidin synthesis; both compounds were not found in this study. Enzymes involved in the pathway are indicated in italics: ANS, anthocyanidin synthase; C3ˈH, 5‐*O*‐(4‐coumaroyl)‐D‐quinate 3′‐monooxygenase; CHI, chalcone isomerase; CHS, chalcone synthase; DFR, bifunctional dihydroflavonol 4‐reductase/flavonone 4‐reductase; F3′5′H, flavonoid 3′,5′‐hydroxylase; F3′H, flavonoid 3′‐monooxygenase; F3H, naringenin 3‐dioxygenase; HCT, shikimate *O*‐hydroxycinnamoyl transferase; UFGT, anthocyanidin 3‐*O*‐glucosyltransferase. (B) KEGG pathway enrichment analysis. Gene expression profiles of 30 unique transcripts in anthocyanin biosynthetic pathway, based on RPKM values. Candidate genes with log_2_RPKM > 2 in at least one sample (columns 1, 2, and 3; independent *N. x* *ventrata* pitcher samples harvested on three consecutive days) are represented.

The biosynthetic pathways to anthocyanins are well known, starting with the shikimate pathway, followed by the phenylpropanoid pathway and different possible related routes within the flavonoids biosynthetic pathways leading to the anthocyanidins cyanidin, pelargonidin, and delphinidin and to their respective anthocyanin glycosides [29, 30, 31] (Fig. 4A). Most of the biosynthetic enzymes are employed in the generation of the anthocyanidins. This was evident in the transcriptome analysis of the corresponding genes, all of which are constitutively expressed (Fig. 4B), suggesting that the biosynthetic pathways for anthocyanins are active, indicated also by the permanent red coloration. Thus, both molecular evidence (a complete set of constitutively expressed anthocyanin biosynthetic genes) and analytical chemistry‐based evidence now demonstrates the existence of anthocyanins in *Nepenthes*.

Although this result is not surprising and was expected, the final proof of anthocyanins in Nepenthaceae was still pending and furthermore supports the recent results for the Droseraceae [12]. We also searched the LC‐ESI‐HRMS datasets for molecular ion peaks [M + H]^+^ of known betalains, but did not detect any betalain‐corresponding peak (Table 1). Moreover, among the enzymes necessary for betalain biosynthesis we only found three transcripts for two basic enzymes related to DOPA metabolism, for example, an weakly expressed aromatic‐*L*‐amino‐acid/L‐tryptophan decarboxylase (K01593; GenBank Acc MZ322092) that may generate dopamine, and two 4,5‐DOPA dioxygenase transcripts (*DODA*) (K15777; GenBank Acc MZ322091, MZ322092); no other transcripts of betalain biosynthesis‐related genes (*CYP76AD1, CYP76AD6, 5GT, 6GT*) were detected. Although the expression of *DODA* sounds interesting as it may result in the formation of betalamic acid, homologs of *DODA* have been found in many anthocyanin generating taxa within angiosperms [3]. Strikingly, a deep search for betalamic acid was not successful (Table 1) suggesting that the enzyme was not built or it remained inactive.

Hence, it can be postulated that at least all carnivorous plants of the taxon Nepenthales contain anthocyanins rather than betalains. From an ecological and economical point of view, the absence of nitrogen‐containing pigments such as betalains makes sense. In particular carnivorous plants that catch insects in order to supplement nutrients with additional nitrogen derived from digested prey [32], should not consume the limited nitrogen for betalain synthesis when anthocyanins might very likely perform similar functions. Nevertheless, not all functions of the red coloration in carnivorous plants are known. The coloration could have initially developed as an adaptive trait, because anthocyanin accumulation is often associated with stress responses [16]. At the same time, it increased prey capture efficiency of the traps by providing attractive visual signals. As insect prey capture rates positively correlate with levels of red pigmentation, it might enhance the trap efficiency by the red color itself or by providing a special background for better recognition [16]. Concerning herbivores, anthocyanins may protect the tissue from attack by herbivores, which are attracted by green color [19, 33]. Moreover, anthocyanins have antioxidant activities which might protect the plant against reactive oxygen species [33]. The strong red pigmentation of the peristome in all *Nepenthes* species supports these hypotheses. Here, more studies need to be done.

## Conflict of interest

The authors declare no conflict of interest.

## Author contributions

AD‐L and AM designed the study. AD‐L and MR performed experiments. AD‐L, MR, DW, HV, and AM analyzed and interpreted the results. AD‐L and AM wrote the manuscript. All authors revised and approved the final version and agree to be held accountable for the article content.

## Supporting information

**Table S1**. Anthocyanin biosynthesis gene index and GenBank accession numbers.

## Data Availability

Additional supporting information may be found online in the Supporting Information section at the end of the article. GenBank accession numbers are free from Jan 1^st^, 2022, and available upon request.
